# Observations of fine coronal structures with high-order solar adaptive optics

**DOI:** 10.1038/s41550-025-02564-0

**Published:** 2025-05-27

**Authors:** Dirk Schmidt, Thomas A. Schad, Vasyl Yurchyshyn, Nicolas Gorceix, Thomas R. Rimmele, Philip R. Goode

**Affiliations:** 1 National Solar Observatory, Boulder, CO USA; 2 National Solar Observatory, Makawao, HI USA; 3 Big Bear Solar Observatory, New Jersey Institute of Technology, Big Bear City, CA USA

**Keywords:** Solar physics, Astronomical instrumentation

## Abstract

Resolving fine structures in the Sun’s corona may provide key insights into rapid eruptions and the heating of the corona. Adaptive optics systems have been used for over two decades to reach the diffraction limit of large telescopes, thereby compensating for atmospheric image blur. Current systems, however, are still limited to observations of the solar disk and fail with coronal objects, leaving fundamental coronal dynamics hidden in that blur. Here we present observations with coronal adaptive optics reaching the diffraction limit of a 1.6-m telescope to reveal very fine coronal details. Furthermore, we discovered a short-lived, fast-moving, finely twisted feature occurring during the decay phase of a flare that quickly destabilized. Coronal adaptive optics increased the spatial resolution by an order of magnitude at visible wavelengths. We report here a large portion of off-limb coronal rain material with observed scales below 100 km. This new adaptive optics scheme opens opportunities for observational discoveries at small scales beyond the solar limb in the highly dynamic corona by exploiting the diffraction limit of large ground-based telescopes.

## Abstract (web summary)

High-resolution observations of fine structure in the Sun’s corona hint at plasma features on the order of 10 km. A new adaptive optics system, Cona, lifts the veil of atmospheric blur over a fast-moving, twisted plasma stream as it becomes unstable.

## Main

Prominences, loops and rain are picturesque plasma phenomena occurring in the Sun’s corona. Understanding the structuring and the evolution of coronal plasma on small scales has been intricately linked to solving the long-standing mysteries of the solar atmosphere^1^: (1) How is plasma in the corona heated to millions of kelvins when the Sun’s surface is only 6,000 K? (2) How and when are eruptions triggered? These eruptions can cause beautiful auroras on Earth but also harm power grids, communication infrastructure and spacecraft.

One avenue for coronal heating is the production of nanoflares in braided coronal loops^2,3^, which have been demonstrated in laboratory plasma experiments^4^. The actual and observational scales in the solar atmosphere and those in the laboratory, however, all differ by several orders of magnitude^5^. Further, coronal rain has been proposed as a marker for coronal heating mechanisms^6^. Although the most advanced space-based imaging instruments have hinted at coronal substructures, even braid-like features^7^, the fundamental structure of the magnetically confined coronal plasma remains poorly understood^8^.

Resolving the fine structure in coronal plasma at visible wavelengths requires large telescope apertures well in excess of 1 m. The resolution of solar structure in such telescopes, however, is naturally not limited by diffraction but by turbulent air in Earth’s atmosphere, an effect known as ‘seeing’^9^. Adaptive optics that corrects for seeing became available for observations of the Sun in the late 1990s. Today, adaptive optics is routinely used for high-resolution observations of the Sun at all major solar telescopes^10,11^, but it is still limited to observations of the solar disk. As a result, valuable radiative diagnostics at the diffraction limit of large apertures have not been available for material and fields beyond the solar limb.

In solar adaptive optics, the shape of an adaptive mirror is adjusted using a correlating Shack–Hartmann wavefront sensor. Such sensors analyse the disturbance of an image of solar structure in a small field (for example, ref. ^11^). As the turbulence in Earth’s atmosphere depends on the viewing direction, in an effect known as anisoplanatism, the position of the field of view of the wavefront sensor must be close to and, ideally, coincide with the target of the observation^12^. Consequently, for coronal observations beyond the solar disk, the wavefront sensor must be able to use structures in the corona, such as prominences, rain and other plasma features. This is where existing solar wavefront sensors fail because they are designed for photospheric structures, such as the omnipresent granulation. The appearances and brightnesses of photospheric and coronal features are vastly different and require distinct wavefront sensors.

Although the technology, parameters and algorithms for a wavefront sensor are well established for the photosphere (for example, ref. ^11^), they are not for coronal features. Low-order adaptive optics correction for prominences was demonstrated at the 0.76-m Dunn Solar Telescope^13^ in a proof-of-concept experiment. The wavefront sensor used all the hydrogen-α light (Hα, 656.3 nm) of a prominence, where it is brightest, while the prominence was observed in the calcium ii line (854.2 nm). We continued to develop coronal adaptive optics at the 1.6-m Goode Solar Telescope (GST)^14,15^, which is the world’s second largest solar telescope.

We report here the implementation and scientific results of mature high-order adaptive optics for objects in the corona, including prominences, loops and rain. This system, called Cona, reached the diffraction limit of the GST for observations in Hα, giving a revolutionary resolution better than 70 km (0.1 arcsec). Within the first days ‘on-sky’, we discovered an unexpected feature exhibiting previously unobserved structures and dynamics. We further found that cooled coronal material exhibits structure down to the diffraction limit of the GST, indicating that the actual scales are much smaller.

## Results

### Coronal adaptive optics of the GST

We built a new wavefront sensor for Hα structures in the corona outside the solar disk for the 1.6-m GST. Over the course of several dozen on-sky testing days over several years, we optimized this wavefront sensor by iterating critical design parameters, such as subaperture size, wavelength band-pass and field of view (both optical and for digital correlation). The noise characteristics, frame rate and quantum efficiency of the wavefront sensor detector also play essential roles and needed to be optimized to achieve high-order, diffraction-limited wavefront correction. This sensor controls an adaptive mirror and uses 50% of the Hα light, where the local plasma is brightest, leaving 50% for scientific instruments, which we captured with a Fabry–Pérot imaging spectrograph, namely the visible imaging spectrometer (VIS) mounted at the GST. We provide more details in Methods.

**Fig. 1:**
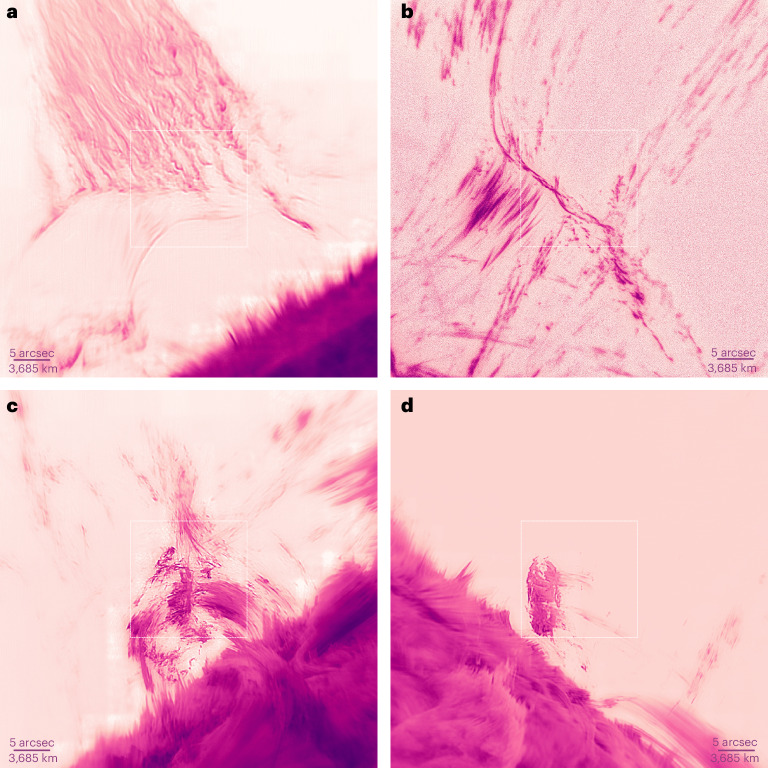
Examples of diverse Hα features outside the solar disk imaged with adaptive optics correction. **a**, Quiescent hedgerow prominence (18:44:13 on 28 May 2024; 1.05 *R*_⊙_, 350.44°). **b**, Loop structures at the base of an erupting filament showing fine braiding in the centre and coronal rain emanating from the top right (17:09:52 on 14 July 2023; 1.03 *R*_⊙_, 253.14°). **c**, Dynamic prominence with large-scale twist alongside raining coronal material (18:16:13 on 28 May 2024; 1.03 *R*_⊙_, 112.91°). **d**, Dense and cool quiescent prominence with complex internal flows (20:01:36 on 29 May 2024). Each frame is about 37,500 km wide (52 arcsec). The squares in the centre of the frames mark the wavefront sensor field of view (16 arcsec). All images were acquired with the VIS instrument at the Hα line centre at the GST. **a**, **c** and **d** were processed with speckle reconstruction^45^, which improved the signal-to-noise ratio. Visible tiles are artefacts of this reconstruction and occur where the local mean brightnesses were incorrectly restored. In **b**, the mean of eight dark-flat-calibrated-only frames were further processed with multiscale Gaussian normalization^47^ to display the dim coronal rain along with the bright loop structure. Evidence of material at the diffraction limit in raw adaptive-optics-corrected images is shown in Extended Data Fig. 1. Artificial colourization was used, so that darker colours are brighter intensities. Images are full frame but oriented for visual appeal. Supplementary Videos 1 and 2 are animations of **c** and **d**. Scale bars are 0.2 arcsec thick.

Figure 1 shows only a small selection of Hα features beyond the solar limb that the coronal adaptive optics has been able to resolve. The image quality of the quiescent hedgerow prominence in Fig. 1a is readily comparable to near-ultraviolet images from the Hinode space telescope in calcium ii at 396.8 nm, which has a diffraction limit of 164 mas (refs. ^16,17^). In our case, the diffraction limit was 85 mas at 656.3 nm. Figure 1b,c clearly resolves fine plasma twisting at arcsecond and subarcsecond scales as well as dim raining coronal material. Figure 1d resolves a dense, cool quiescent prominence showing smooth rapid internal convection-like flows while it hovers closely above the limb.

Although we present Hα images here, the science instruments could have observed at other wavelengths of interest, for example 854.2 nm (calcium ii). In fact, we also took Doppler data at 1,083 nm (helium i) with the near-infrared imaging spectropolarimeter mounted on the GST. The coronal adaptive optics is capable of operating at larger distances from the limb than shown in these examples. The system is limited only by the lack of sufficient Hα structures and by the pointing range of the telescope.

### Discovery of a twisted coronal plasmoid

**Fig. 2:**
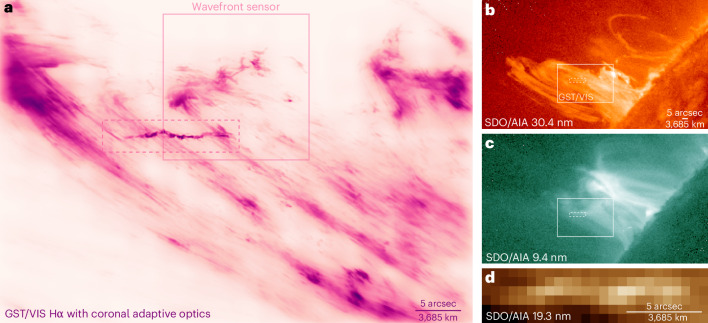
Twisted plasmoid in a post-flare coronal loop system resolved with adaptive optics. **a**, High-resolution view in Hα by the GST using coronal adaptive optics showing plasma at 5,000 to 30,000 K. The plasmoid (1.07 *R*_⊙_, 81.34°) is highlighted with the dashed rectangle and shown enlarged in Fig. 3. **b**, Context view by SDO/AIA in 30.4 nm showing plasma at 50,000 K and the failed eruption material. The rectangles in **b** and **c** mark the area of the image in **a**. **c**, Context view by SDO/AIA in 9.4 nm showing the decay-phase flare emission at 6 million kelvin. **d**, Close-up view of the twisted plasmoid by SDO/AIA in 19.3 nm. AIA has a resolution of 1.5 arcsec. This image is like what the Hα image would look like without adaptive optics at good seeing (*r*_0_ = 9 cm). The field of view of the wavefront sensor is indicated in **a**. The structure seen by this sensor is slightly different because of the wider Hα band-pass in this device. Images were taken between 18:51:47 and 18:51:53 on 18 July 2023. Artificial colourization was used, and darker colours in **a** are brighter intensities. Supplementary Video 3 is an animation of this figure.

While we were patrolling the corona for small-scale Hα features, on 18 July 2023, we stopped at the highly dynamic post-flare loop system shown in Fig. 2 and began to observe the decay of a failed prominence eruption. Hoping to capture tiny structures that demonstrate the performance of the adaptive optics, we became astounded witnesses of the strange fine-structured and fast-evolving plasma feature highlighted in Fig. 2a. The failed eruption coincided with a weak X-ray flare in an active region (GOES class C6.3, NOAA 13379).

**Fig. 3:**
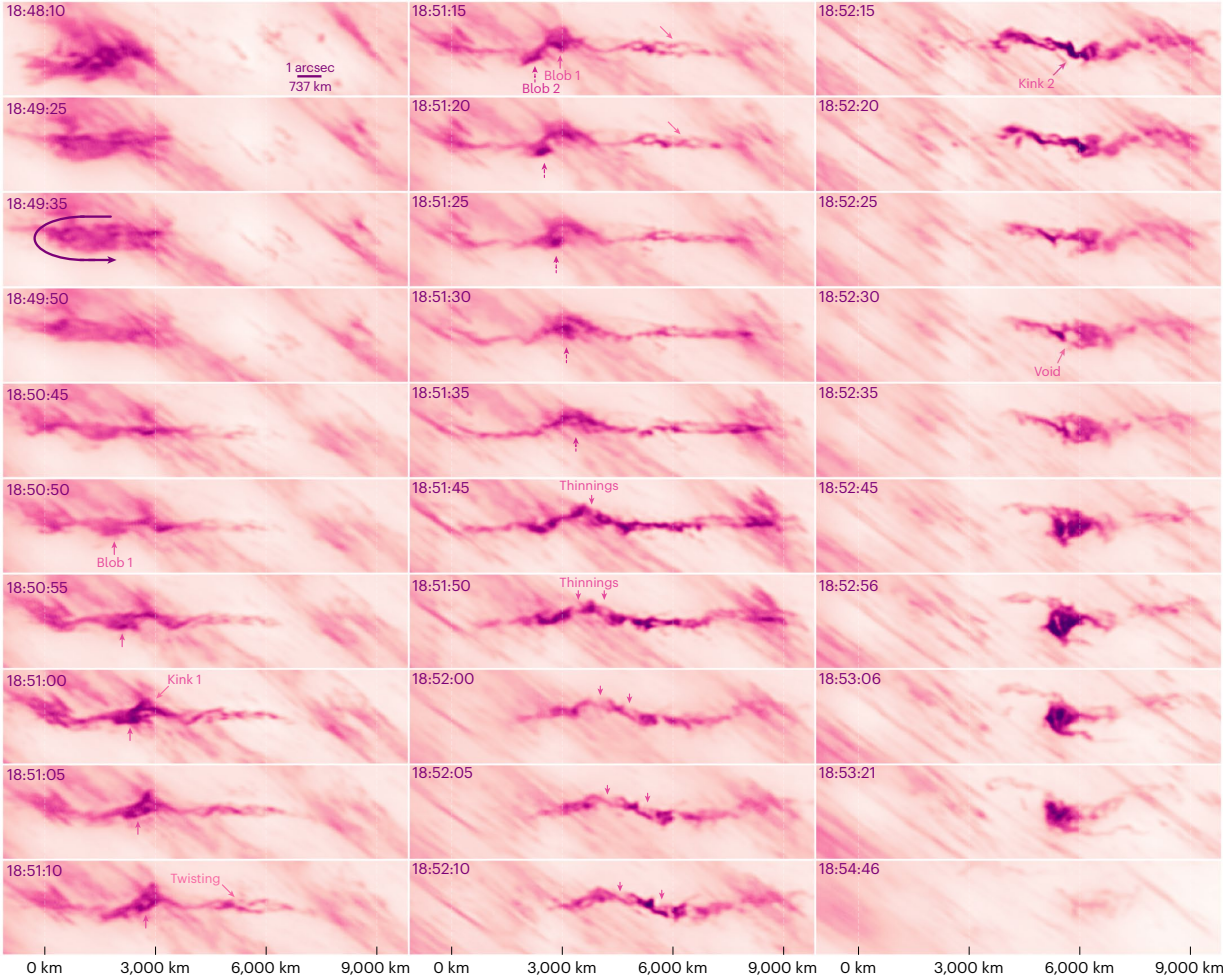
Temporal evolution of the twisted plasmoid. Plasma had accumulated in an ~3,000-km-wide area by 18:48. Around 18:49:35, it seemed that the plasma was structured in a folded stream when the upper half moved left and curled sharply into the lower half, which moved right. While the feature straightened out, its multi-threaded substructure became visible at its head. At 18:50:50, the first blob appeared and moved slightly diagonally. Around 18:51, twisting of the threads became apparent, and a kinked deflection formed while the blob approached it. The kink first remained almost still and then moved right while a second blob appeared at a similar position, also diagonally moving at about 70 km s^−1^. The second blob crossed the kink at around 18:51:45. This feature had a maximum length of ~9,000 km. At 18:51:50, the tip of the kink seemed to be fragmented into three pieces. At 18:52:10, the part right of the 6,000-km mark suddenly stopped moving and later seemed to bounce back while the tail crashed into it and formed a brighter kink at 18:52:15. A void where we saw no Hα formed at the heart of the brightening plasma collision and seemed to be squeezed while some material from the tail of the feature seemed to be diverted below the collision. Other tail material bounced back above the collision, leaving a picture that reminds us of a spiral galaxy with two arms. The feature then dissolved and became invisible in Hα at 18:54. Artificial colourization was used, so that darker colours are brighter intensities. Supplementary Video 4 is an animation of this figure. The structure just left of the kink at 18:52:15 is used in Extended Data Fig. 1 to demonstrate the resolution at the diffraction limit. Scale bar is 64 km tick.

Figure 3 documents the evolution of the feature from a seemingly unstructured plasma cloud that formed near the apex of the post-flare loops into a narrow plasma stream, which dissolved after its front half suddenly stopped and collided with its rear half. The left and right motion of the upper and lower halves of the cloud in the beginning (which is best seen in the animation in Supplementary Video 4) leads us to believe that we observed a plasma stream that made some kind of a turn from a shallow angle.

**Extended Data Fig. 1:**
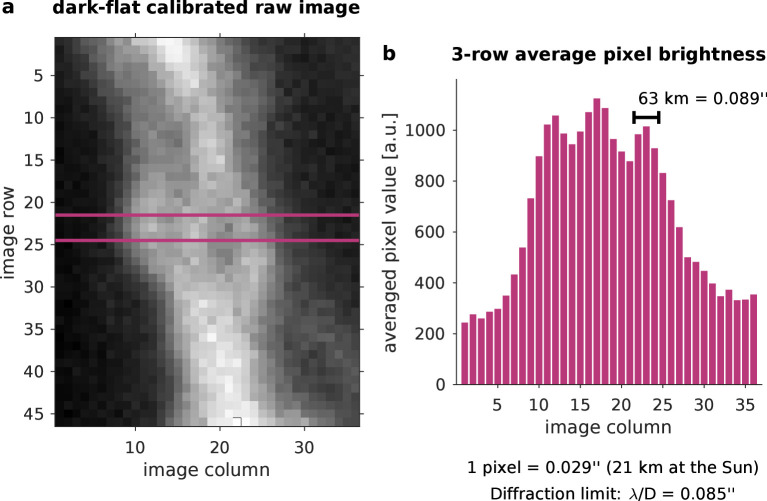
Coronal adaptive optics diffraction-limited imaging without reconstruction. **a**, Close-up of a single dark-flat calibrated image, i. e. prior to speckle reconstruction, of a substructure within the twisted plasmoid at 18:52:15. The structure (rotated) is located just left of the kink indicated by the arrow in Fig. 3. This image is representative of the best images in the burst. **b**, Averaged image brightness of the three pixel rows between the two horizontal lines in the image showing distinct peaks of about 3 pixels width, corresponding to 63 km. This is very close to the theoretical full width at half maximum of the diffraction-limited, idealized Airy disk of 1.029 *λ*/*D* = 0.087 arcsec of the GST. The native widths in this feature are not known but non-zero and contribute to the apparent width after the imaging with the point-spread-function.

The twisted character of the stream is recognizable and distinct from the structure of the background raining loop system. It propagated towards the solar limb at an angle of incidence of about 30°, with its leading edge travelling from the 6,000 to the 9,000-km mark, where it suddenly stopped, in about 55 s corresponding to about 55 km s^−1^. Between 18:51:35 and 18:52:10, the tail of the feature travelled roughly from the 0 to the 3,000-km mark, corresponding to a speed of the order of 80 to 90 km s^−1^. The lateral envelope of the feature measured between 150 and 350 km. The twisted strands were less than 100 km across with structures at the diffraction limit (Extended Data Fig. 1). Although it is difficult to unambiguously quantify the apparent twist, the 18:51:20 sample in Fig. 3 indicates that there was one full wind of narrow threads over a length of ~1,200 km. Two locations within the feature show evidence for a kink-like instability where the material was displaced laterally along its primary axis. The formation of the first kink coincided with the approach of a faster, diagonally moving blob, but it remains speculative whether there was a connection between the two. A second, similar blob coincided with the apparent separation of this kink into fragments with thinner connections. As with its creation, the forces causing the stream to stop and collapse are not immediately apparent.

**Extended Data Fig. 2:**
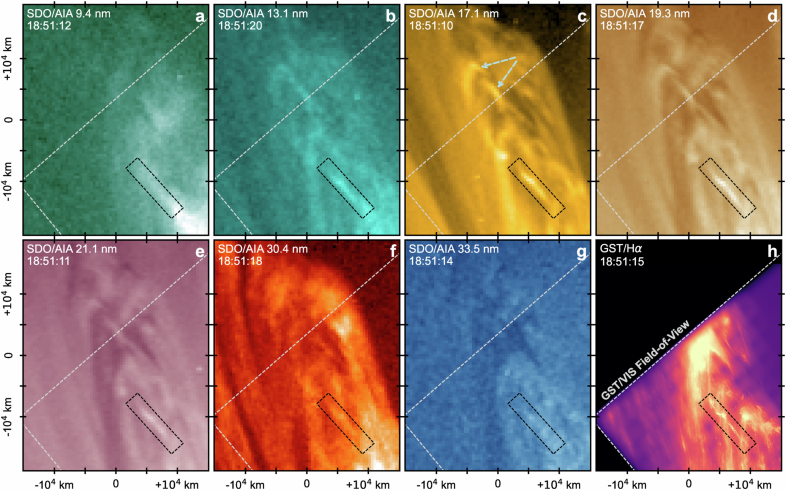
Neighboring features of the observed twisted coronal plasmoid. **a**–**h**, All SDO/AIA extreme ultraviolet bandpass images zoomed into the region just above the twisted plasma structure reported within the GST VIS H*α* data shown in **h** as outlined by the diagonal rectangular box. We call attention to similar plasmoid or blob-like features occurring at this same time and marked with arrows in panel **c**. (An animation of this figure is available online.).

This feature was also captured by the atmospheric imaging assembly (AIA)^18^ onboard NASA’s Solar Dynamics Observatory (SDO)^19^, where it appeared bright in all extreme-ultraviolet channels. However, at only 1.5-arcsec resolution and 12-s cadence, the feature was coarse and inconspicuous and probably did not trigger any interest by scientists. AIA data are complementary to our high-resolution data because they probe different temperature regimes and give insights into the large-scale coronal magnetic environment where the feature developed (Fig. 2b–d). AIA also revealed that the feature was accompanied by other dynamic bright blobs outside our field of view (Extended Data Fig. 2).

This feature, which we refer to as a ‘coronal plasmoid’, is intriguing because its dynamics are distinct from any previously observed chromospheric material embedded in the corona. Similar structures and evolution have also not been observed in the lower chromosphere. As such, the coronal adaptive optics has revealed a unique feature that merits further study.

### Width distribution of coronal rain strands

The underlying distribution of structures in the outer solar atmosphere is unknown at the diffraction limit of the GST, which the adaptive optics promises to resolve. This means that analysing the fine details resolved by coronal adaptive optics is not only a test for the performance of the system but also provides new insights into the existence and distribution of small features in the corona.

**Fig. 4:**
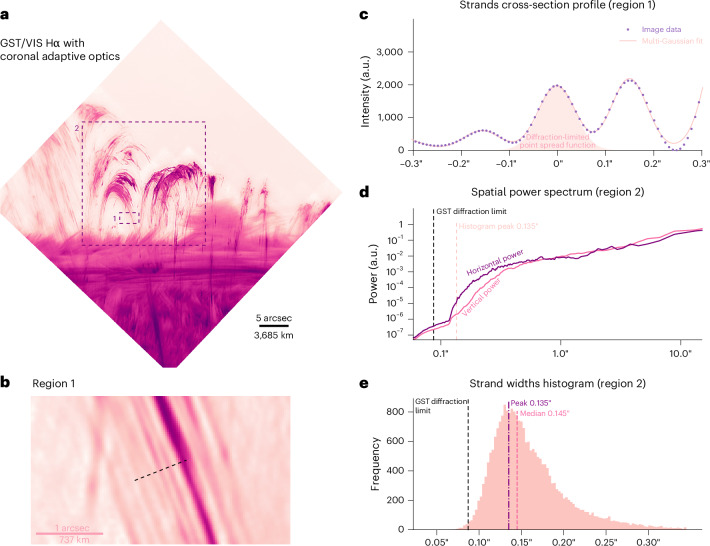
Strand widths in post-flare coronal rain. **a**, Post-flare coronal loops (NOAA 13697; 17:34:30 on 29 May 2024; 1.03 *R*_⊙_, 109.94°; oriented with the radial direction upwards). **b**, Zoom-in of region 1 in **a** with a cross section across narrow features. **c**, Multi-Gaussian fits to the observed intensity along the cross section in **b**, compared to a Gaussian diffraction-limited point spread function. **d**, Power spectra of the GST observations in directions parallel and vertical to the solar limb. **e**, Histogram of fitted strand widths using several additive Gaussian profiles to all off-limb points as described in Methods. Supplementary Video 5 is an animation of **a**.

On 29 May 2024, we targeted the finest scaled coronal phenomena observed to date: coronal rain^20,21^. The field of view of the wavefront sensor was placed near the apex of post-flare coronal loops approximately 18 arcsec above the solar limb (Fig. 4a). The finest rain strands were in region 1, which is shown enlarged in Fig. 4b. A sample cross section across the narrowest strands was selected, and the corresponding intensity profile is shown in Fig. 4c. By fitting several additive Gaussians to this profile, we derived the full-width at half-maximum of the central strand, which appeared at or near the diffraction limit of the telescope of 1.029*λ*/*D* = 87 mas (64 km), where *λ* = 656.3 nm is the Hα wavelength and *D* = 1.6 m is the telescope diameter. This implies that the native width of the strand was much narrower and could have been of the order of 10 km or less.

We derived the distribution of the observed strand widths using two analysis tools. First, we calculated the mean power spectral density within region 2 (Fig. 4a) over the 25-min duration of these observations (Supplementary Video 5). Figure 4d reveals enhanced power, parallel to the solar limb, down to approximately 0.11 arcsec, consistent with the above finding; however, the bulk of the power was between 0.2 and 0.5 arcsec. The rain strands often coherently formed within a larger loop structure, and there was notable power at larger scales.

The second analysis used the fitting method demonstrated in Fig. 4c but applied in a semi-automated fashion to all strands. We first isolated the structures with image segmentation and then derived their orientation using the rolling Hough transform (RHT; Methods)^22^. The resulting histogram in Fig. 4e indicates a peak in the width distribution near 0.135 arcsec (100 km) as observed, coinciding with the diffraction limit of a 1-m aperture telescope. Furthermore, the median value indicates that half of the strands in region 2 are narrower than 0.145 arcsec.

## Discussion

Enabling adaptive optics corrections for observations of features in the Sun’s corona outside the solar disk is a transformative progress. Having achieved an angular resolution better than 0.1 arcsec, the coronal adaptive optics system Cona can successfully probe the finest detail observed beyond the solar limb to date. With the twisted coronal plasmoid, the system enabled the discovery of an unknown phenomenon—which may be the product of small-scale magnetic reconnection—within its first days on-sky. The system further provided new insights into the smallest scales of coronal rain, a marker for coronal heating.

The highly dynamic plasmoid exhibited fast apparent motion of at least 55 km s^−1^, twisted strands, several locations of kink instability and a short lifetime of less than 5 min. It was formed during the decay phase of a C-class flare near the apex of the associated active region, which showed continued signs of large-scale magnetic restructuring. Furthermore, and as SDO/AIA data revealed, this feature emanated from a region where other bright plasma blobs formed outside of the GST field of view (Extended Data Fig. 2). These characteristics lead us to believe that the phenomenon may be intricately linked to the continuing magnetic reconnection within the current sheet of the flare.

Because magnetic reconnection occurs on subkilometre scales, well below observational resolutions, it can be inferred only by actions occurring on large scales. In three-dimensional magnetic reconnection theory, the tearing instability can develop mini-flux ropes within or near the poorly observed current sheet^23^. The complex interactions of these twisted ropes during fast reconnection have been linked to plasmoid generation adjacent to the reconnection location^24,25^, and recent observations at hot coronal temperatures show plasmoid apparent velocities from 79 to 208 km s^−1^ when viewing the flaring region from above, meaning on the solar disk^24^. These aspects are highly compatible with the behaviour of the twisted feature reported herein. An investigation of the temperatures in this region is planned for future work, especially because Hα emission implies cooler plasma that is not fully ionized. Only recently have kink instabilities in such plasmas been studied numerically^26^, and this event could be a useful example of this process.

Furthermore, we have found the width distribution of coronal rain strands extends beneath previous reports made using conventional, photospheric adaptive optics with the wavefront sensor pointed at the solar disk for rain observed on-disk^21,27,28^ and off-limb^29,30^. These previous observations have supported high-resolution 2.5-dimensional magnetohydrodynamic (MHD) simulations that indicate the size distribution of rain peaks between 100 to 200 km (ref. ^20^), which is near the resolution limit of 1-m telescopes. In our observations, however, we found that approximately half of the features were narrower than 100 km and that the widths cascaded down to the diffraction limit of the GST. Our finding indicates that, indeed, currently missing three-dimensional effects could be necessary to improve the accuracy of self-consistent radiative MHD simulations of coronal rain^20^. Moreover, some 2.5-dimensional and three-dimensional simulations with parameterized heating rates do show a notable number of coronal rain blobs with sizes below 50–100 km (refs. ^31,32^).

The three-dimensional structuring for the coronal magnetic field is poorly understood. Recent three-dimensional radiative MHD coronal simulations have challenged early preconceptions of isolated tube-like structures and, instead, indicate that they may be optical illusions under the ‘coronal veil’ scenario^8^. Corresponding curvilinear coronal structures seen in integrated emission resulting from complex magnetic field distributions have been reproduced by several MHD models^33^. So far, however, observations have failed to provide strong support for one theory over another. Coronal rain may point to both the spatial distribution and to the agent of the coronal heating^6^, and observing rain blobs at high resolution could provide an understanding of the fundamental scales of the corona, at least in its fragmented multi-phase state during instabilities. Recent Solar Orbiter observations have investigated the multi-thermal dynamics of coronal rain at the highest resolution in the extreme ultraviolet^34^. Here, we have presented an analysis of cooler plasma in Hα at a roughly three times finer resolution.

Until now, most chromospheric material in the corona has been observed as being predominantly organized in parallel channels, as in Fig. 4, or in sheet-like features within quiescent prominences, as in Fig. 1a. Features may cross or intersect along the line of sight and further show signs of large-scale helicity (see, for example, Fig. 1c). Although some signs of braiding at coronal temperatures have been reported^7,35^ that may be analogous to braided material created in plasma laboratory experiments^4^, until our observations, there has been little evidence of small-scale twisting or braiding at chromospheric temperatures in the solar corona.

The new availability of adaptive optics for off-disk coronal observations will accelerate the research into processes in the Sun’s outer atmosphere, thus repeating the tremendous success story that on-disk photospheric adaptive optics has enjoyed over the last two decades. Although the most apparent value of adaptive optics is its capability to restore images at the diffraction limit, it also greatly improves the scientific utility of a telescope by providing prolonged periods of much improved resolution, even when seeing conditions do not allow for diffraction-limited image correction. Plasma structures beyond the solar limb can now be observed routinely at resolutions previously available only in rare and unpredictable exceptionally good seeing situations. This means that the chance of capturing rare solar events at unprecedented resolution is greatly improved. This aspect of adaptive optics is particularly important because there is not much data or observations of the Sun’s outer atmosphere at high resolution. Further, coronal adaptive optics concentrates the light of small, weak, blurred features—for example, coronal rain blobs—on fewer pixels, thus improving the signal-to-noise ratio and, hence, their detection probability.

Achieving diffraction-limited resolution of fast processes in the corona poses a challenge to existing science instruments. For the most dynamic events that we have captured, we have seen notable evolution from the beginning to the end of bursts spanning 100 images (about 2.5 s). Plasma speeds of well above 100 km s^−1^ are known to occur in the Sun’s corona, which translates to 1.7 diffraction elements for the 1.6-m GST in Hα. To avoid smearing effects at that speed, all data used for image reconstruction must be acquired within about 0.5 s and accordingly faster for larger apertures.

Our coronal Hα wavefront sensor is a relatively inexpensive device, and we expect that other telescopes will adopt similar devices in the near future. Our team is already working on a respective upgrade for the 4-m Daniel K. Inouye Solar Telescope. High-resolution coronal observations using adaptive optics are set to become the norm at large ground-based solar telescopes by the end of this decade.

Our current implementation of coronal adaptive optics is a classical single-conjugate system and, as such, suffers from anisoplanatism in the Earth’s atmosphere, which limits the corrected field of view. Scientific demand for a larger field will emerge and grow while new research is carried out. Our coronal adaptive optics system thus motivates the costly development of laser-beacon multi-conjugate coronal adaptive optics to expand the image correction. Daytime laser beacons^36,37^ that can be fired near the Sun are needed, because—like bright stars in the night sky and in contrast to the photosphere—suitable coronal features are sparse and by themselves provide limited ‘sky coverage’ for adaptive optics. Using laser guide stars will not only allow for adaptive optics correction in the lower corona, where sufficiently bright material frequently exists, but also further out in the corona where no natural objects exist that could be used for wavefront-sensing. Moreover, this first generation of coronal adaptive optics will enable high-resolution spectral and polarimetric measurements of solar prominences, active coronal loops as well as small-scale jets and spicules, thus providing critical data that are needed to lift the veil on the mysteries of the corona but which have not been available so far.

## Methods

### The GST coronal adaptive optics system Cona

We developed the coronal adaptive optics system for the 1.6-m GST at the Big Bear Solar Observatory in California. The off-axis design of the GST makes it ideal for observations of faint coronal features. The secondary mirror and its mounting structure are not in the light path, which, hence, minimizes scattered light and maximizes the throughput. The clear aperture is also advantageous for adaptive optics because no pupil parts are obstructed by the mounting spider of a secondary mirror. Such spiders reduce the signal-to-noise ratio in obstructed parts of the wavefront sensor. The potential spatial discontinuity (fragmentation) in the pupil caused by spiders may lead to differential piston that is not corrected by the adaptive optics system (for example, ref. ^38^).

The GST is home to several state-of-the-art adaptive optics systems, including the multi-conjugate system Clear that has three deformable mirrors. The new coronal wavefront sensor is a conventional correlating Shack–Hartmann sensor (for example, refs. ^10,11^) and was paired with a 357-actuator deformable mirror located in a pupil image.

For the adaptive optics control, we adapted and enhanced the KAOS Evo 2 control software. This in-house developed software has roots at the GREGOR Solar Telescope^39^. KAOS Evo 2 is used in all GST adaptive optics systems, including the multi-conjugate system Clear^40^. An earlier version of the software was also used in the experiments at the Dunn Solar Telescope in 2013 (ref. ^13^). KAOS Evo 2 will also be used for the second-generation adaptive optics at the Daniel K. Inouye Solar Telescope when it is upgraded with ground-layer and multi-conjugate adaptive optics^41^.

### Hα band-pass filter mounted on the wavefront sensor

A key component in the coronal wavefront sensor is the optical Hα filter. The off-disk Hα spectral radiance is typically less than 20% of the photospheric continuum radiance. Therefore, the filter band-pass must be sufficiently narrow to minimize contributions from photospheric light scattered into the line of sight by Earth’s atmosphere and the telescope optics. We used a custom narrowband, high-transmission interference filter with a band-pass width of 0.05 nm and a peak transmission greater than 50% at 656.335 nm. Maximizing the filter transmission (the throughput to the wavefront sensor) helped to preserve the available flux that can be relayed to the science instrumentation. As in any interference filter, the transmitted band-pass shifts slightly in wavelength with incidence angle. As the band-pass shifts towards the wings of the Hα emission line, the total transmitted light weakens. This creates a radial intensity gradient in the field of view or in the pupil, depending on the position of the filter^15^. We converged to a filter position where the pupil is mostly affected by the band-pass shift to avoid any vignetting effects in the fields of view of the subapertures. The consequent intensity gradient across the pupil implies a non-uniform signal-to-noise ratio because images in the subapertures get darker towards the edge of the pupil. The specifications of the coronal wavefront sensor are listed in Supplementary Tables 1 and 2 and compared to the photospheric on-disk sensors at the GST.

### Wavefront sensor flat-fielding

The band-pass shift is also relevant for the flat-fielding of the wavefront sensor. Because the Hα line is visible on the solar disk in absorption against the black-body radiation of the photosphere, the intensity gradient caused by the shifted band-pass is inverted when pointing the wavefront sensor at the solar disk. Consequently, flat-field images acquired on-disk are not applicable off-disk^15^. If possible, we use flat-field frames that are created by moving the line of sight across large diffuse prominences. This is tricky because it requires the existence of respective prominences that ideally have a similar intensity level, such that the same camera gain settings can be used for the actual target prominence. Although a flat-fielded wavefront sensor typically performs better, we have also often used no flat-field correction successfully. This is possible thanks to the low photo-response non-uniformity of the camera pixels.

### Updating the correlation reference for the wavefront sensor

The reference image for the correlation with the images in the subapertures is selected from a particular subaperture near the pupil centre. The reference image in the KAOS Evo 2 software is a fixed snapshot from a previous moment. As the target structure evolves, the reference image must be updated. This is a standard procedure in solar correlating Shack–Hartmann sensors. For on-disk observations, an update interval of 30–60 s is enough. Here, we used 10 s to be able to keep up with fast dynamics off the solar disk. Some very fast-evolving features require updating on the scale of seconds to prevent the control loop from becoming unstable.

### Performance analysis of the adaptive optics

The KAOS Evo 2 control software can store a variety of internal data for a post-facto performance analysis. We demonstrate the performance of the adaptive optics image correction by analysing a 34-s-long control loop dataset recorded at 18:47 on 18 July 2023, during the observation of the twisted plasmoid. The frame rate in the wavefront sensor was 2,240 Hz, and its exposure time was 405 μs. Unfortunately, the camera gain settings used for these data were lost. Thus, we cannot estimate the actual light level and dark noise in the wavefront sensor at that very moment. For the wavefront reconstruction, we used a minimum mean squared error reconstruction matrix for 241 Karhunen–Loeve modes^42,43^.

The cross-correlation peak calculated by the KAOS Evo 2 software between the prominence images in two different subapertures was well above 95%, indicating minimal image noise. In the AO0308 Mk II wavefront sensor of the GST (Supplementary Table 1), a similar value is obtained when it is pointed at high-contrast photospheric features. When pointed at solar granulation, it is typically between 60% and 80%. Any non-common path aberrations and vibrations, if existing, in the image from VIS installed at the GST were not captured in the analysis presented.

### Strehl ratio after adaptive optics correction

Supplementary Fig. 1 shows that the estimated time-variable Strehl ratio for the residual wavefront error as seen by the wavefront sensor ranges between 20% and 40%. A 20% average Strehl ratio is often considered to be sufficient to recover details near or at the diffraction limit when using image reconstruction techniques such as speckle reconstruction. If image vibration (wavefront tip and tilt) is not included in the estimation, the Strehl ratio is up to 60% to 80%. Image vibration does not contribute to image blur in sufficiently short exposures. Fast and strong image vibration that is not well corrected by the adaptive optics often originates from mechanical vibrations somewhere in the telescope. It is reasonable to disregard such effects in a performance analysis because such vibrations can be considered to be beyond the scope of the adaptive optics system.

The Strehl ratio was estimated using the Maréchal approximation:

1 $$S({t}_{i})\approx\operatorname{e}^{-\sum_{n}{\sigma }_{{{\rm{KL}}}_{n}}^{2}({t}_{i})},$$

where $\sum_{n}{\sigma }_{{{\rm{KL}}}_{n}}^{2}({t}_{i})$ is the sum of the phase variances of all Karhunen–Loeve modes as reconstructed by the control software in control loop cycle *t*_*i*_. The uncorrected higher-order modes were neglected because they do not reduce the Strehl ratio substantially at the presented levels, if assuming a Kolmogorov spectrum. The Strehl ratio was denoised using a 2-s-long moving average.

### Image vibration after the adaptive optics correction

The root-mean-squared residual image vibration at the wavefront sensor was 49 mas, which is approximately 60% of the theoretical diffraction limit at 656.3 nm. The vibration was calculated from the two-dimensional subaperture image shifts ${s}_{x}^{(k)}({t}_{i})$ and ${s}_{y}^{(k)}({t}_{i})$ in the *k*th subaperture at time *t*_*i*_ according to

2 $${S}_{x}({t}_{i})=\frac{1}{K}\sum_{k=1}^{K}{s}_{x}^{(k)}({t}_{i}),$$

3 $${\sigma }_{{S}_{x}}^{2}=\frac{1}{N-1}\sum_{i=1}^{N}{\left\vert {S}_{x}({t}_{i})-\overline{{S}_{x}({t}_{i})}\right\vert}^{2},$$

4 $$\begin{aligned}{\sigma }_\mathrm{S}&=\sqrt{{\sigma }_{{S}_{x}}^{2}+{\sigma }_{{S}_{y}}^{2}}\\ &=\sqrt{{(17\,{\rm{mas}})}^{2}+{(45\,{\rm{mas}})}^{2}}=49\,{\rm{mas}},\end{aligned}$$

where *K* = 256 is the number of subapertures in the wavefront sensor and *N* = 76,000 is the number of time samples.

### Transfer function for the adaptive optics

The closed-loop transfer curve in Supplementary Fig. 1 reveals 0-dB bandwidths of 110 and 124 Hz for tip-tilt and wavefront correction, respectively. These bandwidths are even slightly higher than when observing granulation with the on-disk AO308 Mk II system of GST.

The transfer function for wavefront mode *k* was estimated from

5 $${T}^{\;(k)}(\;f\;)=\frac{{{\rm{PSD}}}_{{\rm{closed}}\,{\rm{loop}}}^{(k)}(\;f\;)}{{{\rm{PSD}}}_{{\rm{open}}\,{\rm{loop}}}^{(k)}(\;f\;)},$$

where ${{\rm{PSD}}}_{{\text{closed loop}}}^{(k)}(\,f\,)$ is the temporal power spectral density of the root-mean-square wavefront error in the *k*th mode as reconstructed from the wavefront sensor measurements in the closed control loop. ${{\rm{PSD}}}_{{\text{open loop}}}^{(k)}(\,f\,)$ is the estimated wavefront error before the correction. It was computed from the closed-loop error plus the deformable mirror commands. The commands from two loop cycles earlier were used to account for the latency in the deformable mirror response. The power spectral density was calculated using Welch’s method with a segment length of 1 s, 50% overlap between segments and apodization of each segment with a Hamming window.

### Seeing conditions

We estimated the Fried parameter *r*_0_ for the seeing in Earth’s atmosphere^44^ to be of the order of 8 to 14 cm for the dataset used in this performance analysis. This estimate is based on fitting the modal spectrum of the reconstructed wavefront to the spectrum of the theoretical Kolmogorov turbulence. It is difficult to obtain a more precise and accurate estimate of *r*_0_ from the available data because the spectra do not fit well. Subjectively, the seeing could be described as ‘normal good seeing’ for the GST.

### Hα science observations

The Hα science data were acquired using VIS, which is a single Fabry–Pérot interferometer offering rapidly tunable narrow band-pass imaging. With its telecentric optical configuration, it achieves a spectral band-pass of 0.007 nm for a resolving power of ~10^5^ at 656.3 nm. To maximize the imaging cadence to resolve the rapid dynamics of the upper atmosphere, we elected to operate VIS using a single wavelength sample near the Hα line centre wavelength. The VIS focal plane was recorded using a pco.panda 4.2 bi camera, which yielded a 77% increase of photon collection efficiency compared to the standard VIS configuration. The detector format is 2,048 × 2,048 pix^2^, and it samples the focal plane with 29 mas per pixel with frame rates up to 40 Hz. At the Hα wavelength, the Nyquist sampling for the 1.6-m telescope aperture was *λ*/(2*D*) = 42 mas, and so the VIS oversampled the diffraction limit by ~45%. That is, the theoretical Airy disk was spread over 3 pixels in diameter. For data acquired in 2023, the exposure times were nominally 5 mas acquired at 37 Hz. For data acquired in 2024, a newer version of this camera was installed that offers a low-light mode with reduced dark noise (1.06 *e*^−^ versus 2.28 *e*^−^). We used the low-light mode in all 2024 observations presented here and lowered the exposure time to 1.6 ms to further mitigate the blurring effect of any non-common-path turbulence or vibrations that may or may not exist between the wavefront sensor and the VIS camera. Images were acquired in bursts of 100 frames with variable timing between bursts depending on the expected dynamic in the scenery.

### Science image processing and speckle reconstruction

We dark-flat calibrated the raw VIS Hα image bursts and then processed them into an average image using the speckle-imaging KISIP software^45^, unless noted differently. Speckle imaging mitigates the effect of residual seeing not corrected by the adaptive optics. We used the generic speckle transfer function model built into KISIP instead of a transfer function calibrated for these data, so that the photometric properties of the object may not be accurately reconstructed in the images. In some bursts, the plasma features moved notably between the beginning and the end of the 100-frame burst. We used only 20 frames of each burst for the speckle imaging to avoid smearing effects in the resulting image. Common practice is to use at least 70 frames for good atmospheric statistics. A camera with a higher frame rate could be a future upgrade and would allow us to use more frames for the reconstruction.

### Image coalignment and registration

Solar adaptive optic systems are designed to stabilize an image based on a reference feature (a non-point-source ‘guide star’). When that feature moves, the system will attempt to keep it at its original position in the image by driving the field-steering tip-tilt mirror. This effect is amplified in the off-disk case by fast-moving dynamic guide features. To compensate for this effect in the data reported here, we used SDO data at extreme-ultraviolet wavelengths and 160 nm for co-registration. Off-limb, the 160-nm channel was dominated by chromospheric emission^46^ and most closely matched features observed in Hα.

### Measuring the widths of coronal rain strands

Semi-automated measurements of coronal rain widths were made by first segmenting the strands and then determining their orientation with the RHT method^22^. Segmentation involved first creating a high-pass filtered image by dividing the original image by a version convolved with a two-dimensional Gaussian kernel (*σ* = 3 pixels). The resulting image was then binarized according to a 5% filtered intensity threshold. By using an RHT kernel size of 31 pixels and a threshold RHT resultant length of 0.85, we identified only pixels within the image that have linear coherence in one direction on a length scale of ~0.9 arcsec. This limited the size of structures extracted to those narrower than ~0.3 arcsec. For strands wider than 1 pixel, this process identified several pixels across the structure. To reduce the sampling to 1 pixel along an individual cross section, we found the corresponding pixel in the direction perpendicular to the strand axis that has the largest intensity in the original image. We then performed several Gaussian fits to intensity profiles extracted within ±0.35 arcsec in the direction perpendicular to each strand axis. The minimum intensity was subtracted from the profile to approximate the local background intensity, which includes atmospheric and instrumented scattered light. Each fit used five Gaussian profiles added together. The central Gaussian, which targets the strand being measured, was constrained such that it was separated from the neighbouring Gaussian profiles by at least 5 pixels. The root-mean-square width of the profiles was constrained to be less than 5 pixels. We further required that the derived central position of the strand was within 1 pixel of the maximum intensity of the strand. These safeguards ensured that the central strand was not overfitted by several blended profiles. In total, we extracted the widths of the strands in region 1 (Fig. 4) as fitted from 40,128 individual strand cross sections.

## Supplementary information

Supplementary Information Supplementary Tables 1 and 2 and Fig. 1.

Supplementary Video 1 Video for Fig. 1c. Dynamic prominence with large-scale twist alongside the raining coronal material.

Supplementary Video 2 Video for Fig. 1d. Dense and cool quiescent prominence with complex internal flows.

Supplementary Video 3 Video for Fig. 2. Twisted plasmoid in the post-flare coronal loop system resolved with adaptive optics and compared to SDO/AIA images.

Supplementary Video 4 Video for Fig. 3. Temporal evolution of the twisted plasmoid.

Supplementary Video 5 Video for Fig. 4a. Post-flare coronal rain. The video was digitally stabilized to compensate for drifts and rotation.

Supplementary Video 6 Video for Extended Data Fig. 2 showing neighbouring features of the observed twisted coronal plasmoid.

Supplementary Data 1 Speckle reconstructed frame.

Supplementary Data 2 Average of 8 dark-flat calibrated frames.

Supplementary Data 3 Speckle reconstructed frame.

Supplementary Data 4 Speckle reconstructed frame.

Supplementary Data 5 Speckle reconstructed frame.

Supplementary Data 6 Speckle reconstructed frame for 18:52:15.

Supplementary Data 7 Speckle reconstructed frame.

Supplementary Data 8 Dark-flat calibrated frame.

## Data Availability

GST/VIS Hα data used in Figs. 1, 2a, 3 (18:52:15) and 4 and Extended Data Fig. 1 are available in Supplementary Data files 1–8. All data needed to evaluate the conclusions of this study about the evolution of the coronal plasmoid (Fig. 3) are presented in this publication. Images from NASA’s SDO instruments are publicly available through https://sdo.gsfc.nasa.gov/data/. KAOS Evo 2 telemetry data is proprietary and can be made available upon reasonable request for the purpose of reproducing the analysis.

## Code availability

The KISIP speckle processing^45^ software is freely available at https://gitlab.leibniz-kis.de/kisip/kisip-woeger. The coronal rain analysis included the use of Python-based tools developed by T.A.S. in the context of ref. ^22^. The code can be made available upon reasonable request for the purpose of reproducing the analysis.
