# Supplementary material for: Observations of fine coronal structures with high-order solar adaptive optics
Source: Nat Astron. 2025 May 27;9(8):1148–57. doi: 10.1038/s41550-025-02564-0 (PMC12360950; doi:10.1038/s41550-025-02564-0)
Supplement: Supplementary file 1 — Supplementary Tables 1 and 2 and Fig. 1. [file 41550_2025_2564_MOESM1_ESM.pdf]

nature astronomy

Supplementary information https://doi.org/10.1038/s41550-025-02564-0

Observations of fine coronal structures with high-order solar adaptive optics

In the format provided by the authors and unedited

Supplementary Table 1 Coronal versus photospheric wavefront sensors. Comparison of key specifications of the wavefront sensors used at the Goode Solar Telescope for coronal adaptive optics (Cona), and for classical single-conjugate (AO308 MK II) as well as multi-conjugate adaptive optics (Clear) for the photosphere.

Adaptive optics system	Cona (coronal, Hα)	AO308 Mk II (photospheric)	Clear (photospheric)
Type	Correlating Shack-Hartmann	Correlating Shack-Hartmann	Correlating Shack-Hartmann
Field of view	16×16 arcsec	12.4×12.4 arcsec	35×35 arcsec with 3×3 subfields
Subaperture size	8.8 cm	8 cm	8.8 cm
Number of subapertures	256	308	208
Number across pupil diameter	18	20	16 (stopped-down)
Maximum frame rate	2240 Hz	1750 Hz	1567 Hz
Read-out window	400×400 pixels	416×416 pixels	992×992 pixels
Exposure time (typical)	400 µs	<200 µs	<200 µs
Wavelength (0° incidence)	656.335 ± 0.05 nm	525 ± 25 nm	525 ± 25 nm
Nyquist sampling	0.77 arcsec / pixel	0.68 arcsec / pixel	0.62 arcsec / pixel
Pixel scale	0.80 arcsec / pixel	0.62 arcsec / pixel	0.60 arcsec / pixel
Correlation size	20×20 pixels	20×20 pixels	20×20 pixels
Camera model	First Light Imaging C-Blue One 0.5 MP	Adimec Q-2HFW	Mikrotron EoSens 3CXP

Supplementary Table 2 Cameras for coronal and photospheric wavefront sensing. Comparison of key specifications of the solar wavefront sensor cameras in the Goode Solar Telescope.

Camera model	First Light Imaging C-Blue One 0.5 MP	Adimec Q-2HFW	Mikrotron EoSens 3CXP
Sensor model	Sony IMX 426	CMOSIS CSI2100	Cypress LUPA3000
Sensor type	CMOS	CMOS	CMOS
Electronic shutter type	global	global	global
Quantum efficiency at 656 nm	≈ 60%$^{(*)}$	≈ 48%$^{(*)}$	37 % (at 680 nm)$^{(*)}$
Dark noise	40 to 2.8 e$^{-(**)}$	≈ 1000 e$^{-(*)}$	21 e$^{-(*)}$
Saturation capacity	23,500 to 248 e$^{-(**)}$	≈ 2,000,000 e$^{-(*)}$	27,000 e$^{-(*)}$
GST wavefront sensor(s)	Cona	AO-308 Mk II, Turbulence Profiler	Clear (MCAO), Ground-layer AO

$^{*)}$ per manufacturer data sheets, $^{**)}$ per in-house EMVA1288 testing, lowest to highest gain settings with 8-bit readout

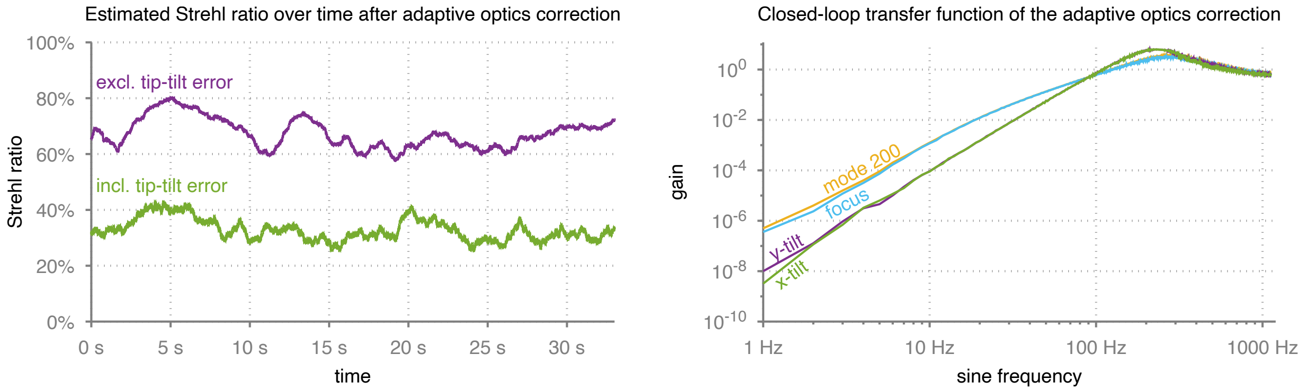

Supplementary Figure 1 Performance metrics for the adaptive optics correction. The estimated Strehl ratio after adaptive optics correction as seen by the wavefront sensor over 34 seconds on July 18, 2024, 18:47, and the closed-loop transfer function of the same time representing the rejection of the temporal power spectrum of the indicated wavefront-error mode.
